# Military Personals

**Published:** 1918-10

**Authors:** 


### MILITARY PERSONALS

Dr. Francis M. O'Gorman who has received the commission of Captain in the M. R. C. and who has orders to report to a camp in Alabama this month, was given a dinner of 40 places at the Weyand Cafe in Buffalo.

Maj. William G. Bissell of the 65th regiment of the state guard has been appointed by Adjutant General Sherrill as instructor in hygiene, first aid and sanitation at the instruction camp for officers of the state guard to be held at Camp Whitman the first two weeks of September.

Dr. Ross Nairn has been commissioned Captain in the M. R. C. and Drs. Glenn Arthurs, John Finnegan, Howard Ludwig and Walter Zielinski were given Lieutenants' commissions.

To Camp Custer, Maj. T. H. McKee, Buffalo.

To Camp Devens, Lieuts. J. P. Jeffman, Buffalo; R. D. Richman, Morton.

To Camp Dodge, Capt. K. E. Williams, Rome.

To Camp Jackson, Capt. M. E. Leary, Rochester.

To Camp Jos. E. Johnston, Lieut. D. J. Tillen, Elmira.

To Camp McClellan, Capt. F. M. O'Gorman, Buffalo.

To Camp Meade, Capt. T. I. Townsend, Binghamton.

To Fort Des Moines, Major T. Wright, Buffalo.

To Fort Ontario, Lieut. W. L. Wooden, Clifton Springs.

To Lakewood, N. J., Capt. J. B. Ringland, Oswego.

To New Haven, Conn., Lieut. D. Yunj, Buffalo.

To Plattsburg Barracks, Capt. J. G. Stowe, Buffalo.

To Rockefeller Institute, Capt. W. M. Halsey, Oswego.

To Washington, Capt. H. L. Raymond, Collins; Major J. R. Gaylord, Buffalo.

To Wichita Falls, Texas, Capt. S. A. Munford, Ithaca.

Honorably discharged on account of physical disability existing prior to entrance into service, Capt. F. W. Filsinger, Buffalo; Capt. W. J. Aubry, Cohoes.

To Camp Crane, Capt. J. C. Davis, Rochester.

To Camp Gordon, Lieut. T. L. McNamara, Corning.

To Camp Hancock, Capt. C. O. Sayres, Rochester.

To Fort Oglethorpe, Lieuts. R. L. Cooley, E. J. Ludwig, J. E. Trudnowski, Buffalo; Capt. F. S. Winslow, Rochester; Lieuts. J. E. Wright, Menden; J. B. Deuel, Rochester.

To Camp Dix, Lieut. C. S. Laird, Westfield.

To Camp Green, Capt. F. W. Kennedy, Rochester.

To Camp Sevier, Capt. G. S. Skiff, Gainesville.

To Camp Shelby, Lieut. S. D. Earhart, Clifton Springs.

To Camp Wadsworth, Lieut. F. M. Kujawa, Buffalo.

To Fort Benjamin Harrison, Capt. Ross Nairn, Buffalo; Lieut. H. G. Hotchkiss, Rochester.

